# Development of a transoral robotic surgery program in Canada

**DOI:** 10.1186/1916-0216-42-8

**Published:** 2013-02-01

**Authors:** Anthony C Nichols, Kevin Fung, Corina Chapeskie, Samuel A Dowthwaite, John Basmaji, Sandeep Dhaliwal, Christopher CT Szeto, David A Palma, Julie A Theurer, Martin A Corsten, Michael Odell, John W Barrett, Jason H Franklin, John Yoo

**Affiliations:** 1 Department of Otolaryngology - Head and Neck Surgery, Western University, London, ON, Canada; 2 London Regional Cancer Program, London, ON, Canada; 3 Lawson Health Research Institute, London, ON, Canada; 4 Department of Oncology, Western University, London, ON, Canada; 5 Department of Pathology, Western University, London, ON, Canada; 6 Department of Otolaryngology – Head and Neck Surgery, University of Ottawa, Ottawa, ON, Canada; 7 Department of Otolaryngology - Head and Neck Surgery, Victoria hospital, London Health Science Centre, Room B3-431A, 800 Commissioners Road East, London, ON, N6A 5W9, Canada

**Keywords:** Transoral robotic surgery, Head and neck cancer, Canadian healthcare

## Abstract

Due to significant differences in healthcare structure between the United States and Canada, there are unique barriers to adopting new medical technology in Canada. In this article, we describe our experience developing a transoral robotic surgery (TORS) program at Western University. Specifically, we outline the steps that were necessary to obtain institutional and multidisciplinary team approval, financial support, as well as surgeon and allied healthcare personnel training. This experience can potentially be used as a roadmap for other Canadian institutions pursuing a TORS program.

## Introduction

Head and neck squamous cell carcinoma (HNSCC) is the fifth most common cancer in Canada, affecting more than 4500 Canadians each year [1]. Despite a national decrease in rates of alcohol and tobacco use, there has been an epidemic rise in the incidence of one subset of HNSCC - cancers arising from the oropharynx [2,3]. Over the last three decades, oropharyngeal cancers have tripled, an increase now known to be due to oral infection with the human papillomavirus (HPV) [3,4]. At many institutions, chemoradiation is the standard of care. However it carries significant short and long term side effects, such as mucositis, fibrosis, dysphagia, xerostomia, and tissue necrosis [5-7]. These results have led the oncology community to reconsider surgical approaches in a minimally invasive context. Since receiving FDA approval in 2009, transoral robotic surgery (TORS) for the treatment of T1-2 oropharyngeal and laryngeal cancers has become rapidly adopted at many institutions in the United States. The initial results of many small studies suggest that this surgical approach provides excellent survival rates and superior speech and swallowing outcomes relative to other organ preservation approaches [8-10]. However, prior to December 2010 a TORS program had not been developed in Canada. Delayed adoption of this promising technology is at least in part due to the disparate healthcare systems. The aim of this article is to describe the development of a TORS program in Canada, highlighting the challenges that needed to be overcome, some of which are unique to a universal healthcare system.

## Process of introducing TORS at the London health sciences centre

### Multidisciplinary team collaboration and evidence based treatment planning

Treatment of all head and neck cancer patients at the London Regional Cancer Centre is conducted within the framework of the Cancer Care Ontario guidelines [11]. Treatment recommendations are by consensus of our multidisciplinary team including radiation oncologists, medical oncologists, and head and neck surgeons. To implement any significant changes to the treatment practices of our institution, we required the approval of the multidisciplinary team. We therefore began with a multidisciplinary journal club to review the relevant literature regarding transoral resection and chemoradiation in an attempt to compare the results of the two treatments. These discussions were supplemented by educational visits by international experts who have been instrumental in developing TORS programs at their respective institutions. One clinical expert was a radiation oncologist with extensive experience in providing post-operative radiation for patients who have undergone a TORS procedure. He outlined the treatment fields he used in order to de-intensify post-operative radiation and highlighted the excellent functional outcomes that had been achieved including low gastrostomy tube (G-tube) rates of approximately 2% [8-10]. The second expert was a head and neck surgeon who has utilized TORS for over five years.

After several discussions, a protocol meeting was arranged to discuss the possible integration of TORS into our current treatment algorithms. Our review of current practices revealed that following FDA approval, many centers in the United States began using TORS as the preferred modality of treatment for oropharyngeal and laryngeal cancer including those with advanced regional metastases. The majority of these patients still require post-operative adjuvant therapy [8-10]. In order to select for patients who could avoid adjuvant therapy entirely, our consensus was to adopt a more conservative approach, offering surgery to selected stage I-II oropharyngeal and supraglottic cancers. Other eligible patients include those with absolute surgical indications, such as T1-2 radiation failures and small salivary gland tumors, where the treatment alternative would traditionally require open surgery. We also developed evidence-based guidelines for post-operative chemotherapy and radiation following TORS.

### Institutional approval

In order to obtain ethical and administrative approval for TORS there were a number of challenges that had to be overcome related to the novelty of treatment, costs, and operating room requirements. The Canadian Surgical Technologies & Advanced Robotics (CSTAR) Centre, an organization that supports all minimally invasive surgery (MIS) research at our institution, played an important role in facilitating the approval of this pilot project. CSTAR provides ethical review and funding for approved projects in order to help overcome the unique difficulties in generating research in the field of MIS in Canada. This organization has established a formal application process for new procedures that requires certificates of training, a description of the procedure in question, study designs that include patient outcome measures, and a budget calculated with a cost per case assessment. After a formal review by the MIS Committee, our application was approved.

### Training/Certification

Presently, there are only three TORS training programs in North America, located in Philadelphia, Pennsylvania; Sunnyvale, California; and Houston, Texas. These sites offer one-week certified programs that include animal, cadaveric and observational training for head and neck surgeons. Access to these training programs can be challenging as there is an excess of demand with only a limited number of training spots. Moreover, the producers of the da Vinci robot, Intuitive Surgical, Inc., have partial oversight of the selection of surgeons accepted into the program. Intuitive Surgical, Inc. has set strict criteria for entry including evaluation of the institution and surgeon’s case volumes, access to a robot and the ability of the surgeon to perform the first robotic procedure within 2 weeks of course completion. This last requirement can be particularly challenging due to the short time frames involved with treating patients with HNSCC. Ultimately, two Head and Neck surgeons from the London Health Sciences Centre (ACN and KF) were accepted in to the program and completed training at the University of Pennsylvania.

### Study design

The multidisciplinary team stressed the importance of prospective trials and to collect follow-up data on specific surgical and quality of life outcome measures. Subsequently, we delineated outcome measures for our preliminary experience including margin status, blood loss, complication rates, gastric tube rates and post-operative days in hospital. Other outcome metrics included functional and quality of life (QOL) outcomes such as validated QOL questionnaires, modified barium swallows, and vocal acoustic analysis [12-14]. These tests would be administered pre-operatively and at three, six and 12 months post-operatively.

### Funding and hospital resources required

The cost of da Vinci robot and its maintenance is significant (Table 1). The cost of the new SI version of the robot is $2.5 million. In addition, there are annual maintenance fees and costs for disposable instruments used in each case. In the United States insurance providers do not cover the additional cost incurred by the robotic components, so the institution must absorb it. This cost is recouped by capturing additional patients by this high-profile technology. Increased patient volume results in greater use of hospital services and consultations, and subsequently increased institutional revenues.

**Table 1:** Cost of da Vinci robot purchase, annual maintenance and, initial and per case costs specific to trans-oral robotic surgery

**Expenditure**	**Costs specific to trans-oral surgery cases**		**Costs of operating the da Vinci Robot**^··^	
	**Initial costs**	**Cost per case**^·^	**Initial cost**	**Annual cost**
da Vinci-SI robot			$2,500,000.00	
Maintenance Contract				$112,000.00
FK Retractor (Olympus)	$10,735.00			
Maryland Dissector (20 uses)	$7,106.00	$355.30		
Cautery Instrument (18 uses)	$6,394.00	$355.22		
Cautery Spatula Tip (10)	$1,465.00	$146.50		
**Total**	**$25,700.00**	**$857.02**	**$2,500,000.00**	**$112,000.00**

^·^ Costs per case specific to TORS as quoted by Minogue Medical 2010:

^··^ Costs of purchasing and maintaining the da Vinci Robot system were not specific to TORS procedures and thus were not incurred by the head and neck department.

In Canada, this strategy is incompatible with our healthcare system hospital funding structure. With hospitals placing a premium on cost-containment, there is no financial incentive for patient recruitment. Among the priorities of the London Health Sciences Centre has been to evaluate technological advances, especially in the field of minimally invasive surgical (MIS) care. Our institution in conjunction with private donors established funding for MIS initiatives, which enabled the purchase of the necessary capital and disposable instruments. The average cost per case of expendable equipment is $857.02 USD (See Table 1 for a cost breakdown). Since the da Vinci Robot is used at our institution by various specialties, the purchase and maintenance costs of the robot were not borne by the Otolaryngology-Head and Neck Surgery department.

Once diagnosed, HNSCC needs to be treated within a short time frame (one to three weeks) in contrast to other robotic procedures that can be completed on a less urgent basis. This is particularly true in the oropharynx and larynx where tumour growth can change the extent of surgery, which in turn can affect oncologic and functional outcomes. Thus once a case is identified, access to the surgical robot must be available within a reasonable time frame. We worked closely with hospital leadership to obtain assurances that all cancer cases could be completed within two weeks of the decision to proceed with surgery.

### Collaboration with anaesthesia

Like other upper aerodigestive tract surgeries, airway management remains critical to the welfare of the patient and is facilitated through effective communication between the surgeon and the anaesthesiologist. Unlike open surgery, patients undergoing TORS do not typically require a tracheostomy [10]. However, airway management was discussed with the anaesthesia service in a preliminary meeting to ensure adequate precautions would be taken with these patients. Airway considerations are reviewed with the consultant anaesthesiologist prior to each case. Specific recommendations were made with respect to pre-operative steroid use, the use of an un-taped armoured endotracheal tube, and continuous post-operative pulse oxygenation monitoring.

### Nursing and allied health education

A great deal of effort was undertaken to ensure adequate nursing and allied heath team training to support the TORS program. Educational materials and training sessions were provided in the pre-operative clinic and on the floor. Pre-operative considerations included patient counseling and arranging consultations with anesthesia and medicine. Post-operative nursing care education emphasized airway concerns, bleeding risk, and speech and swallowing evaluation. For operating room nurses, a full day training session was coordinated. In addition, a Minogue Medical (distributor of the da Vinci robot in Canada) biomedical engineer is present for all cases to assist with technical difficulties and provide further training.

### Developing a transoral robotic surgery program at other Canadian institutions

There are 13 Canadian hospitals that have da Vinci surgical robots and thus the potential to start a TORS program (Table 2). There are three models including the Standard, S and Si. The S and Si are ideal for TORS due to the lower profile arms which allow easier access to the oral cavity.

**Table 2:** da Vinci Robot Sites in Canada

**Institution**	**Robot model**
CHUM - St. Luc (Montreal)	Si
Hôpital du Sacré-Cœur de Montréal	Standard
Humber River Regional Hospital (Toronto)	Si
Jewish General Hospital (Montreal)	S
London Health Sciences Center	Si
Montreal General Hospital	Si
RockyView Hospital (Calgary)	Si
Royal Alexandra Hospital (Edmonton)	S
St. Joseph’s Hospital (Hamilton)	Si
St. Michaels Hospital (Toronto)	S
The Ottawa Hospital	Si
Toronto General Hospital	S
University of Alberta Hospital (Edmonton)	S
Vancouver General Hospital	S

Information obtained from Minogue Medical Inc.

All the above mentioned institutions in collaboration with Minogue Medical have developed a rigorous credentialing process to ensure safe implementation of all new robotic procedures. The prerequisites to complete this process include completion of the surgeon clinical pathway (training course), appropriate surgeon and nursing onsite training, appointment of a robotic coordinator with the appropriate training, and facilities for instrument cleaning and sterilization. Currently the University of Ottawa is following this pathway with the goal of starting their TORS program in early 2013.

## Future directions

To date, the oncologic and functional outcomes of TORS have been encouraging. However, this technology has not been evaluated in randomized controlled trials. We have recently opened a phase II randomized trial of primary TORS versus radiation for early stage oropharyngeal cancer named ORATOR (Carcinoma of the Oropharynx: Radiation versus Transoral Robotic Surgery) that is funded by the Canadian Cancer Society [15]. This study is powered to detect a clinically significant superior swallowing outcome in the TORS arm using the MD Anderson dysphagia inventory (MDADI) [13]. In addition, there are two multi-centred trials being planned in the United States through the ECOG and RTOG cooperative groups studying HPV-positive and negative oropharyngeal cancer, respectively. This level of evidence is necessary for ultimate validation of this surgical approach and broader adoption in Canada and beyond.

## Competing interests

Dr. Anthony Nichols has a research grant from Merck Canada Inc.

This material has never been published and is not currently under evaluation in any other peer-reviewed publication.

The following grants provided support for this project: London Health Sciences Foundation.
